# Supplementary material for: Contactless cardiac arrest detection using smart devices
Source: NPJ Digit Med. 2019 Jun 19;2:52. doi: 10.1038/s41746-019-0128-7 (PMC6584582; doi:10.1038/s41746-019-0128-7)
Supplement: Supplementary file 1 — Supplemental material [file 41746_2019_128_MOESM1_ESM.pdf]

	Gender	Age (yrs)	Recording period (mins)	Hypopneas	Central apneas	Obstructive apneas	AHI
Patient 1	Female	58	456	16	52	3	9.3
Patient 2	Male	46	464	26	157	11	25.1
Patient 3	Female	60	471	113	1	45	20.3
Patient 4	Male	59	462	105	8	112	29.2
Patient 5	Female	57	466	30	3	15	6.2
Patient 6	Male	68	435	6	2	6	1.9
Patient 7	Male	37	442	2	0	0	0.3
Patient 8	Male	35	373	80	33	88	32.4
Patient 9	Female	59	291	57	18	9	17.3
Patient 10	Female	93	304	20	16	6	8.3
Patient 11	Male	56	393	21	1	11	5.0
Patient 12	Female	50	441	20	0	5	3.4
Mean (range)		57 (35-93)	416 (291-471)	41 (2-113)	24 (0-157)	26 (0-112)	13 (0.3-32.4)

Supplementary Table 1. Demographic summary of sleep study patients with number of hypopnea and apnea events as well as the apneas-hypopneas index (AHI)
